# Subjective Workload and Cognitive Failure Among Emergency Nurses: A Cross‐Sectional Mediation Analysis of Job Content and Job Burnout

**DOI:** 10.1002/nop2.70846

**Published:** 2026-09-21

**Authors:** Zahrasadat Abedi, Keyvan Hosseingholipour, Parisa Nazmi, Naser Parizad

**Affiliations:** ^1^ Student Research Committees Urmia University of Medical Sciences Urmia Iran; ^2^ Department of Medical Surgical Nursing, Nursing & Midwifery School Urmia University of Medical Sciences Urmia Iran; ^3^ Department of Nursing Imam Reza Hospital Urmia Iran; ^4^ Patient Safety Research Center, Clinical Research Institute Urmia University of Medical Sciences Urmia Iran

**Keywords:** burnout, cognitive dysfunction, emergency service, hospital, nursing staff, occupational stress, professional, workload

## Abstract

**Aims:**

To examine associations between subjective workload and cognitive failure among emergency nurses and assess whether job content and job burnout statistically mediate these associations.

**Background:**

Cognitive failure in nurses is associated with occupational and psychological demands and may compromise patient safety and well‐being. Examining the relationships among subjective workload, job‐related factors, and cognitive failure may inform management interventions in emergency care.

**Design:**

A descriptive, cross‐sectional correlational study.

**Methods:**

The study population comprised 320 eligible emergency nurses in Urmia. Using proportional quota sampling based on the number of nurses in each hospital, 295 nurses were recruited. Ultimately, 255 completed questionnaires were included in the final analysis. Data were collected using a demographic questionnaire, the Broadbent Cognitive Failures Questionnaire, the NASA Task Load Index (NASA‐TLX), the Maslach Burnout Inventory, and the Karasek Job Content Questionnaire. Data analysis was conducted using SPSS version 26 and SmartPLS version 4.

**Results:**

Subjective workload was positively associated with cognitive failure (*β* = 0.478, *p* < 0.001), job content (*β* = 0.577, *p* < 0.001) and job burnout (*β* = 0.425, *p* < 0.001). Job content was positively associated with cognitive failure (*β* = 0.241, *p* < 0.001) and job burnout (*β* = 0.278, *p* < 0.001), whereas job burnout was not significantly associated with cognitive failure (*β* = −0.030, *p* = 0.639). Job content showed significant indirect associations between subjective workload and cognitive failure (*β* = 0.139, *p* = 0.001) and between subjective workload and job burnout (*β* = 0.160, *p* < 0.001). The indirect association between subjective workload and cognitive failure through job burnout was not statistically significant (*β* = −0.013, *p* > 0.05).

**Conclusion:**

Higher subjective workload was associated with greater cognitive failure among emergency nurses. Job content, but not job burnout, demonstrated a statistically significant indirect association in the relationship between subjective workload and cognitive failure. These cross‐sectional findings should be interpreted with caution, and longitudinal studies are needed to clarify the temporal sequence and examine potential causal pathways.

**Implications for Nursing Management:**

Healthcare organizations should consider strategies to optimize job content, including enhancing workplace support and addressing prolonged or excessive working hours. These strategies may be relevant to addressing cognitive strain and job‐related challenges among emergency nurses and may support staff well‐being and patient safety.

## 1 Background

Nurses routinely work under demanding clinical conditions characterized by high patient acuity, time pressure, complex care coordination and extended working hours, all of which impose substantial physical and psychological demands (Geraghty et al. 2025). These conditions may adversely affect mental health and increase the likelihood of cognitive failure, ultimately compromising care quality and patient safety (Hitchcott et al. 2017). Cognitive failure refers to lapses in perception, memory, or motor functioning that result in errors during routine tasks (Aschwanden et al. 2020). In clinical settings, cognitive lapses may manifest as documentation errors, missed clinical information, reduced concentration and other performance deficits that adversely affect patient care processes (Rodziewicz and Hipskind 2020). Furthermore, occupational stressors and perceived workload have been associated with an increased likelihood of cognitive failure (Parizad et al. 2026).

Subjective workload is a multidimensional construct reflecting nurses' perceptions of the physical, mental and temporal demands associated with task performance (Tubbs‐Cooley et al. 2019). A meta‐analysis reported that 54% of nurses experience high subjective workload, with higher levels observed in developing countries and emergency departments (Yuan et al. 2023). In nursing practice, time pressure, complex patient needs, inadequate staffing and high patient volume have been associated with increased perceived workload and fatigue (Ma et al. 2020; Khanade and Sasangohar 2017). Prolonged exposure to these occupational demands may also be associated with emotional strain, diminished coping capacity and poorer occupational well‐being (Khanade and Sasangohar 2017). Consequently, such conditions have been consistently linked to burnout and impaired work functioning among nurses in demanding healthcare environments (Jin et al. 2023).

Burnout, characterized by emotional exhaustion, depersonalization and reduced personal accomplishment, has consistently been associated with demanding working conditions among nurses (Khan et al. 2025). Persistent workload pressure, role overload and insufficient opportunities for recovery have also been linked to higher levels of burnout (Jun et al. 2021). Burnout has further been associated with difficulties in attention, memory and executive functioning, suggesting that chronic occupational strain may affect cognitive functioning among nurses (Søvold et al. 2021). However, growing evidence indicates that the psychological mechanisms linking occupational stressors to cognitive functioning and well‐being extend beyond burnout (Søvold et al. 2021; Bakker et al. 2023). Recent studies have highlighted the roles of emotion regulation, stress appraisal and psychological flexibility and inflexibility in shaping healthcare professionals' responses to demanding clinical environments. For example, Di Gesto et al. (2025) found that emotion regulation difficulties and psychological inflexibility were associated with poorer well‐being among intensive care nurses, suggesting that psychological processes beyond burnout may also influence nurses' responses to workload (Di Gesto et al. 2025). Although these processes are relevant to nurses' responses to occupational stress, the present study focuses on job content and burnout as theoretically grounded factors associated with subjective workload and cognitive failure.

Within the Job Demands–Resources (JD‐R) framework, job content encompasses the structural and psychosocial characteristics of the work role, including task complexity, role clarity, cognitive demands, emotional demands, coordination requirements and decision‐making expectations (Handel 2017). When these job characteristics are experienced as unfavourable or highly demanding, they may be associated with greater cognitive strain, mental fatigue and attentional lapses (Mehri et al. 2022). Similarly, unfavourable job content may be associated with higher burnout because unclear expectations, limited control and insufficient support are associated with greater perceived strain in daily work (Ren et al. 2024). Furthermore, the impact of these occupational demands often extends beyond the clinical environment and affects nurses' personal lives. Recent evidence indicates that high workloads, extended working hours and irregular shift patterns often conflict with family responsibilities, increasing psychological strain and undermining work‐life balance (Sh 2026; Ghanim and Ibrahim 2026).

Emergency departments are characterized by unpredictability, rapid decision‐making demands and high workload, making emergency nurses particularly vulnerable to cognitive failure and burnout (Surendran et al. 2024). A recent meta‐analysis reported that emergency nurses experience significantly higher subjective workload than other healthcare providers (Yuan et al. 2023). Recent evidence has further reinforced the relevance of these relationships. Cortés‐Álvarez et al. (2024) confirmed associations between burnout and cognitive impairment in hospital nurses (Cortés‐Álvarez et al. 2024), Surendran et al. (2025) highlighted the cognitive burden experienced by emergency nurses (Surendran et al. 2025), and Gündüz and Öztürk (2025) reported that mental workload was significantly associated with burnout among intensive care nurses (Gündüz and Öztürk 2025).

Although previous studies have established associations between cognitive failure, subjective workload, job burnout and job content (Parizad et al. 2026; Kakemam et al. 2019; Jarahian Mohammady et al. 2018), these variables have not been sufficiently examined within a single structural model focused on emergency nurses, particularly with explicit consideration of the potential indirect pathways through job content and burnout. Specifically, limited evidence is available regarding the indirect pathways of job content and burnout in the association between subjective workload and cognitive failure. Examining these associations may provide valuable insights for nursing management to support nurses' well‐being and patient safety. Therefore, this study used structural equation modelling (SEM) to examine the association between subjective workload and cognitive failure among emergency nurses and to determine whether job content and job burnout statistically mediate this association.

### 1.1 Theoretical Framework

This study was guided by the JD‐R model (Bakker et al. 2023) and Cognitive Load Theory (CLT) (Sweller et al. 2019) as complementary frameworks for understanding how work conditions may relate to emergency nurses' cognitive and psychological outcomes. Together, these frameworks provide a structured basis for examining the relationships among subjective workload, job content, cognitive failure and job burnout.

### 1.2 Job Demands–Resources (JD‐R) Theory

The JD‐R theory provides a comprehensive framework for explaining how job demands and job resources interact to influence employee well‐being and performance. Job demands, including high subjective workload and complex job requirements, require sustained physical and psychological effort and are therefore associated with physiological and psychological costs, such as burnout and cognitive failure (Bakker et al. 2023). In emergency nursing settings, these demands include high patient turnover, time pressure, complex clinical decision‐making and limited opportunities for recovery. From a JD‐R perspective, these conditions may be related to energy depletion, emotional strain and impaired occupational functioning (Søvold et al. 2021; Dall'Ora et al. 2020; Keykaleh et al. 2018).

### 1.3 Cognitive Load Theory (CLT)

CLT complements the JD‐R framework by explaining the mechanisms underlying cognitive failure. CLT proposes that working memory has a limited capacity and that excessive cognitive demands can impair essential cognitive processes, including attention, memory and decision‐making (Sweller et al. 2019; Paas and Van Merrienboer 2020; Sewell et al. 2019). In emergency care environments, where subjective workload and task complexity may exceed nurses' cognitive capacity, nurses face an increased risk of cognitive overload. This perspective helps explain why high workload may be associated with cognitive lapses, reduced task performance and difficulty sustaining attention during urgent clinical situations (Parizad et al. 2026; Surendran et al. 2025).

### 1.4 Hypotheses Development

#### 1.4.1 Subjective Workload and Cognitive Failure

Empirical evidence indicates that higher subjective workload is associated with poorer attention, memory and overall cognitive performance among nurses (Jarahian Mohammady et al. 2018; Pérez‐Fuentes et al. 2018). Excessive mental demands may overwhelm available cognitive resources, thereby increasing the likelihood of errors and cognitive lapses (Surendran et al. 2024, 2025; Ciğerci and Özpınar 2026).

*H1: Subjective workload is positively associated with cognitive failure among emergency nurses*.

#### 1.4.2 Subjective Workload and Job Burnout

Extensive research has consistently linked excessive workload to emotional exhaustion and other dimensions of burnout (Xiong et al. 2024; Zanabazar and Jigjiddorj 2022). High cognitive and emotional demands may deplete nurses' coping resources, thereby contributing to higher levels of job burnout (Bakker and Demerouti 2017).

*H2: Subjective workload is positively associated with job burnout among emergency nurses*.

#### 1.4.3 Indirect Association Between Subjective Workload and Cognitive Failure Through Job Content

Psychosocial job characteristics, including job demands, autonomy and role clarity, are closely associated with nurses' cognitive performance (Mehri et al. 2022). Job content was examined as a potential pathway for indirect associations, as workload may be associated with nurses' cognitive and psychological outcomes not only through the intensity of experienced demands, but also through how these demands are structured, controlled and supported within the work environment. In this study, job content was modelled as an overall latent construct to capture the broader psychosocial work environment and reflect the combined effects of multiple interacting job characteristics in high‐intensity emergency settings. Although this theory‐driven approach was appropriate for the study aims, the individual dimensions, including decision latitude, psychological demands and social support, may have distinct associations with burnout and cognitive failure and should be examined in future research.

*H3: Subjective workload is positively associated with job content among emergency nurses*.

*H4: Job content is positively associated with cognitive failure among emergency nurses*.

*Core Hypothesis 1: Job content demonstrates a statistical indirect association in the relationship between subjective workload and cognitive failure among emergency nurses*.

#### 1.4.4 Indirect Association Between Subjective Workload and Cognitive Failure Through Job Burnout

Job burnout has been consistently associated with cognitive impairments, including deficits in memory, attention and executive functioning (Cortés‐Álvarez et al. 2024; He et al. 2017). High subjective workload may also be associated with greater job burnout, which may further be associated with cognitive failure (Gündüz and Öztürk 2025; Abareshi et al. 2022).

*H5: Job burnout is positively associated with cognitive failure among emergency nurses*.

*Core Hypothesis 2: Job burnout demonstrates a statistical indirect association in the relationship between subjective workload and cognitive failure among emergency nurses*.

#### 1.4.5 Indirect Association Between Subjective Workload and Job Burnout Through Job Content

Adverse job content may be associated with higher levels of burnout, particularly when characterized by high demands, low autonomy and inadequate organizational support. These conditions have frequently been associated with emotional exhaustion and other symptoms of burnout (Wong et al. 2024; García‐Sierra et al. 2016; Arthur et al. 2025).

*H6: Job content is positively associated with job burnout among emergency nurses*.

*Core Hypothesis 3: Job content demonstrates a statistical indirect association in the relationship between subjective workload and job burnout among emergency nurses*.

**FIGURE 1:**
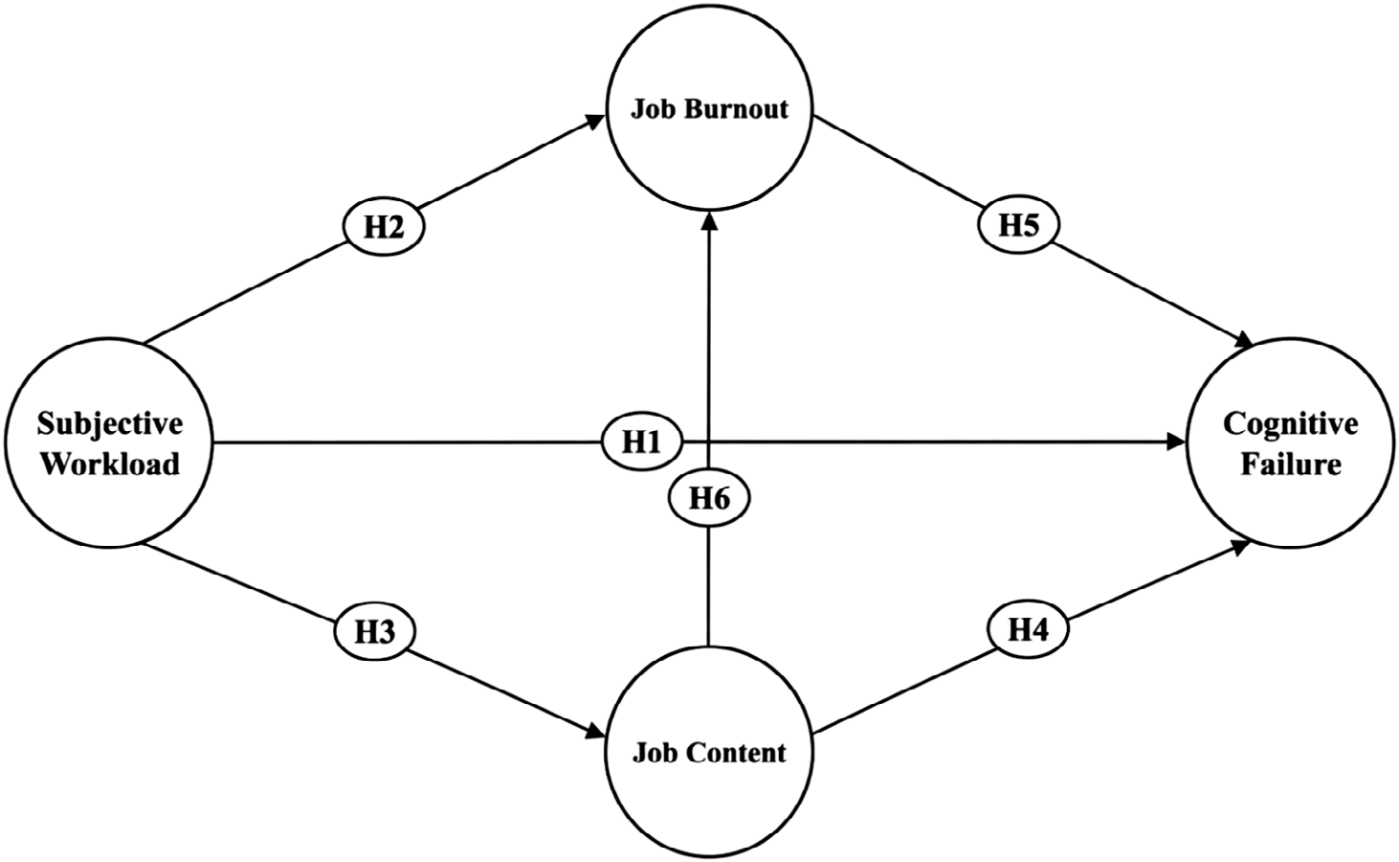
The Conceptual Framework.

The conceptual framework illustrating the study variables and hypothesized associations is presented in Figure 1.

## 2 Methods

### 2.1 Study Design and Setting

This descriptive, cross‐sectional correlational study was conducted using SEM in 2024. The study population consisted of nurses working in the emergency departments of teaching hospitals affiliated with Urmia University of Medical Sciences, Iran. Emergency departments were selected because of their high workload, unpredictable patient flow and complex clinical decision‐making demands. This study was reported in accordance with the Strengthening the Reporting of Observational Studies in Epidemiology (STROBE) guidelines to ensure complete and transparent reporting (Table S1).

### 2.2 Sample Size and Sampling Procedure

The minimum sample size was determined based on the Partial Least Squares Structural Equation Modelling (PLS‐SEM) guidelines recommended by Hair Jr. et al. (2021), using two complementary approaches (Hair Jr. et al. 2021). First, the 10 times rule indicated a minimum of 80 participants, as the Job Content construct had the highest number of indicators (8). Second, an a priori power analysis using G*Power 3.1.9.7, assuming a small effect size (f^2^ = 0.05), *α* = 0.05, and power = 0.80, yielded a minimum sample size of 222 participants.

**FIGURE 2:**
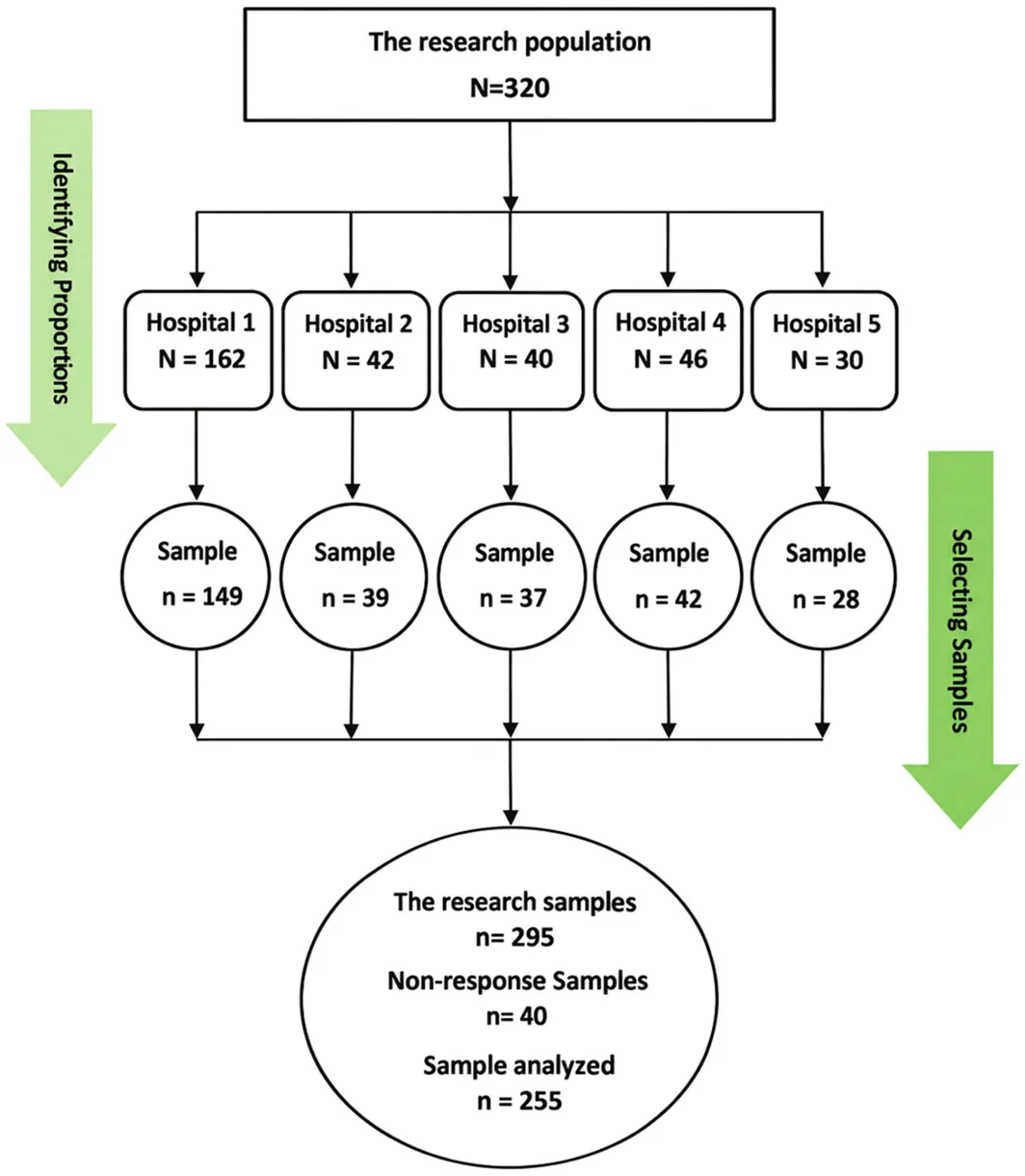
Study sampling flow diagram.

The study population comprised 320 eligible emergency nurses working in the emergency departments of five teaching hospitals. Quota sampling was performed proportional to the number of emergency nurses in each hospital (Hospitals 1–5). Following coordination with department managers, 295 nurses were recruited. Ultimately, 255 completed questionnaires were returned and included in the final analysis, while 40 questionnaires were either not returned or incomplete. This final sample exceeded both minimum sample size requirements and ensured adequate statistical power for the PLS‐SEM analysis. Although this approach improved representation across participating hospitals, the use of nonprobability quota sampling may have limited representativeness and external validity because of potential selection bias. The sampling distribution and flow are shown in Figure 2.

### 2.3 Eligibility Criteria

Inclusion criteria were: (i) at least six months of work experience in an emergency department, (ii) a minimum educational qualification of a bachelor's degree in nursing, (iii) current employment in an emergency department and (iv) willingness to participate. Questionnaires with more than 20% missing responses were excluded from the analysis.

### 2.4 Data Collection Instruments

Data were collected using five standardized instruments:

#### 2.4.1 Demographic Questionnaire

A structured demographic form was used to collect participants' personal and occupational characteristics, including gender, age, education level, marital status, number of children, work experience, income, job position, type of work shift, weekly working hours, employment status and hospital affiliation.

#### 2.4.2 Job Content Questionnaire (JCQ)

Job content was assessed using the validated Persian version of the Karasek Job Content Questionnaire (JCQ) developed by Choobineh et al. This version was translated using forward–backward procedures and psychometrically evaluated among Iranian hospital nurses; therefore, no additional translation was required in the present study (Choobineh et al. 2011). The JCQ comprises eight subscales: job skill (6 items), decision authority (3 items), psychological job demands (5 items), supervisor support (4 items), coworker support (4 items), physical activity (3 items), isometric physical load (2 items) and job insecurity (3 items). Responses are rated on a four‐point Likert scale ranging from 1 (strongly disagree) to 4 (strongly agree) (Karasek et al. 1998). The Persian JCQ has demonstrated acceptable reliability and validity, with Cronbach's alpha coefficients ranging from 0.54 to 0.85 and a reported convergent validity value of approximately 0.75 (Choobineh et al. 2011). In the present study, the JCQ dimensions were specified as formative indicators of job content because they represent distinct demand and resource characteristics that collectively define the psychosocial work environment. To ensure a consistent and intuitive interpretation of this formative construct, the resource‐based indicators, including job skill, decision authority, supervisor support and coworker support, were reverse scored before analysis. Consequently, higher scores on the overall “job content” construct consistently indicated a less favourable psychosocial work environment, characterized by higher job demands and lower job resources.

#### 2.4.3 The National Aeronautics and Space Administration Task Load Index (NASA‐TLX)

Subjective workload was assessed using the NASA Task Load Index developed by Hart and Staveland. The instrument evaluates six dimensions: mental demand, physical demand, temporal demand, performance, effort and frustration. Each dimension is rated on a scale from 0 to 100. The final workload score is calculated using the weighted procedure based on 15 pairwise comparisons; the weighted dimension scores are summed and divided by 15, producing a total score ranging from 0 to 100 (Hart and Staveland 1988; Hoonakker et al. 2011). The validated Persian version developed by Mohammadi et al. was used. In their study, the original instrument was translated into Persian, reviewed by two ergonomics specialists and then back‐translated into English. After comparison with the original instrument and implementation of the necessary revisions, the face validity of the Persian version was confirmed. The Persian version showed acceptable internal consistency, with a Cronbach's alpha of 0.847 (Mohammadi et al. 2013). In the structural model, the six NASA‐TLX dimensions were specified as formative indicators of subjective workload because they represent distinct components that jointly form the overall workload construct.

#### 2.4.4 Maslach Burnout Inventory (MBI)

Job burnout was assessed using the validated Persian version of the 22‐item Maslach Burnout Inventory developed by Moalemi et al. The instrument was translated into Persian by a qualified translator and reviewed against the original version by three medical experts. Because it had previously been validated in Iranian populations, no additional translation was performed in the present study (Maslach et al. 1986; Moalemi et al. 2018). This instrument consists of 22 items across three subscales: emotional exhaustion (9 items), depersonalization (5 items) and personal accomplishment (8 items). Responses are scored on a seven‐point Likert scale ranging from 0 (never) to 6 (every day). Maslach et al. reported Cronbach's alpha values of 0.86 for emotional exhaustion, 0.81 for depersonalization, 0.82 for personal accomplishment, and 0.93 for the overall scale (Maslach et al. 2001). In the Persian context, Cronbach's alpha for the overall questionnaire was 0.75, with values exceeding 0.70 for all subscales (Moalemi et al. 2018).

#### 2.4.5 Cognitive Failures Questionnaire (CFQ)

Cognitive failure was assessed using the 25‐item Cognitive Failures Questionnaire developed by Broadbent et al. (Broadbent et al. 1982). Each item is rated on a five‐point Likert scale ranging from 0 (never) to 4 (almost always), yielding a total score between 0 and 100, with higher scores indicating more frequent cognitive failures. The CFQ evaluates three domains: distractibility, false triggering and forgetfulness (Bannerjee et al. 2025). The validated Persian version developed by Shahgholian was used in the present study. To prepare this version, the original English questionnaire was translated into Persian by the researcher and independently back translated into English by two experts with doctoral degrees in English language studies (Shahgholian 2024).

Previous research has shown strong internal consistency for the CFQ, with Cronbach's alpha values typically ranging from 0.84 to 0.91, and test–retest reliability of 0.82 (Broadbent et al. 1982; Wallace 2004). The Persian version demonstrated acceptable face and content validity, as well as adequate reliability, with Cronbach's alpha coefficients ranging from 0.65 to 0.81 (Shahgholian 2024).

### 2.5 Data Collection Procedure

The researcher visited the emergency departments of the selected hospitals to introduce the study and explain its objectives. Written informed consent was obtained from all participants before data collection. Data were collected in person between April and September 2024. Participants were given one week to complete the questionnaires. All responses were collected anonymously to protect participant confidentiality. Questionnaires with more than 20% missing data were excluded from the analysis. No statistical imputation was performed for excluded questionnaires, and only questionnaires meeting the predefined completeness criterion were retained.

The dataset analysed in the present study was derived from the same primary data collection as a previously published article (Parizad et al. 2026). However, the present manuscript constitutes a distinct secondary analysis with different objectives, hypotheses, and analytical approaches. The previous study examined direct associations among subjective workload, job content, burnout and cognitive failure using standard regression analyses. In contrast, the present study is grounded in the JD‐R model and CLT and evaluates a comprehensive mediation model. Specifically, it examines whether subjective workload is indirectly associated with cognitive failure through job content and job burnout. The study's novel contribution is the use of PLS‐SEM to test this theoretical framework and examine potential psychological and occupational mechanisms beyond direct associations. Thus, the present analysis provides a distinct conceptual and methodological contribution from the previous publication.

### 2.6 Data Analysis

Data were analysed using IBM SPSS Statistics for Windows, version 26 (IBM Corp., Armonk, NY, USA), and SmartPLS version 4 (SmartPLS GmbH, Bönningstedt, Germany). Quantitative variables were presented as mean ± standard deviation, whereas categorical variables were reported as frequencies and percentages.

The measurement models were evaluated according to whether the constructs were specified as reflective or formative. Cognitive failure and job burnout were modelled as reflective constructs. Their measurement properties were assessed using indicator loadings, Cronbach's alpha, Dijkstra–Henseler's rho (rho_A), composite reliability (rho_C), average variance extracted (AVE), and discriminant validity. Subjective workload and job content were modelled as formative constructs because their distinct dimensions were assumed to jointly form the respective constructs. The formative measurement models were evaluated using outer weights, outer loadings, the statistical significance of the outer weights based on bootstrapping, and variance inflation factor (VIF) values to assess collinearity among the formative indicators.

Discriminant validity among reflective constructs was assessed using the Fornell–Larcker criterion and the heterotrait–monotrait ratio (HTMT). The structural model was evaluated using path coefficients (*β*), t statistics, explained variance (R^2^), effect sizes (f^2^), and predictive relevance (Q^2^predict). Mediation was examined using bootstrapping based on standardized indirect effects. Potential demographic and occupational confounders, including age, years of work experience, shift work and employment type, were included as control variables in the PLS‐SEM model. Direct paths from these covariates to cognitive failure and job burnout were estimated. For the remaining statistical tests, a two‐sided *p*‐value of less than 0.05 was considered statistically significant.

### 2.7 Ethics Statement

This study was conducted in accordance with the principles of the Declaration of Helsinki. Ethical approval was obtained from the Ethics Committee of Urmia University of Medical Sciences (Ethics code: IR.UMSU.REC.1403.226). Written informed consent was obtained from all participants prior to data collection. Participation was voluntary, and responses were collected anonymously to ensure confidentiality.

The primary data collection for this study was approved under the aforementioned ethics code. Although a portion of this dataset was previously used to examine direct associations between job demands, resources, and cognitive failures (Parizad et al. 2026), the present study was specifically designed to address distinct research questions concerning the indirect associations involving job content and burnout. The analytical approach, PLS‐SEM, and the theoretical frameworks applied in the present study were not reported in the earlier publication, thereby minimizing the risk of redundant or overlapping publication of the same findings.

## 3 Results

The total research population comprised 320 eligible emergency nurses across five teaching hospitals. Using quota sampling proportional to the hospital populations, 295 nurses were selected to participate. Of these, 255 completed questionnaires were included in the final analysis, while 40 questionnaires were not returned or were incomplete. The specific reasons for nonresponse were not recorded. Incomplete questionnaires were excluded from the analysis. All 255 questionnaires included in the final analysis contained complete data, and no data imputation was performed. The participants' mean age was 35.00 ± 7.50 years, and their mean work experience was 10.39 ± 7.44 years. The mean subjective workload, cognitive failure, job content, and job burnout scores were 51.98 ± 11.32, 46.85 ± 19.98, 154.47 ± 13.64, and 66.87 ± 13.70, respectively.

Regarding marital status, 155 participants (60.8%) were married and 100 (39.2%) were single. Most participants were female (67.1%), whereas 32.9% were male. Most participants held a bachelor's degree (94.1%), while 5.9% had a master's degree. A total of 204 nurses (80.0%) worked rotating shifts, and 51 (20.0%) worked fixed shifts. Employment types included contractual (36.5%), temporary (20.4%), and permanent (43.1%). Additional demographic characteristics are presented in Table 1.

**TABLE 1:** Demographic characteristics of the study participants.

Variable	Category	*n*	%
Marital status	Single	100	39.2
	Married	155	60.8
Gender	Female	171	67.1
	Male	84	32.9
Education level	Bachelor's degree	240	94.1
	Master's degree	15	5.9
Work shift	Fixed (morning/evening)	51	20.0
	Rotating shift	204	80.0
Employment status	Contractual	93	36.5
	Temporary	52	20.4
	Permanent	110	43.1
Income status	Income<expenses	227	89.0
	Income = expenses	25	9.8
	Income>expenses	3	1.2
Position	Nurse	247	96.9
	Head nurse	8	3.1

*Note:* Values are presented as frequency (*n*) and percentage (%).

### 3.1 Measurement Model Assessment

The measurement model was assessed according to the reflective or formative specification of each construct. For the reflective constructs, all indicator loadings were statistically significant and exceeded the recommended threshold. Indicator loadings ranged from 0.978 to 0.985 for cognitive failure and from 0.736 to 0.885 for job burnout. Cognitive failure demonstrated satisfactory internal consistency and convergent validity, with Cronbach's alpha = 0.981, rho_A = 0.982, rho_C = 0.988, and AVE = 0.964. Job burnout also demonstrated acceptable measurement properties, with Cronbach's alpha = 0.715, rho_A = 0.790, rho_C = 0.836, and AVE = 0.631 (Table 2).

**TABLE 2:** Reflective measurement model assessment.

Construct	Indicator	Indicator	Cronbach's α	rho_A	rho_C	AVE
Cognitive failure	Distractibility	0.982	0.981	0.982	0.988	0.964
	False Triggering	0.978				
	Forgetfulness	0.985				
Job burnout	Personal accomplishment	0.753	0.715	0.790	0.836	0.631
	Emotional exhaustion	0.885				
	Depersonalization	0.736				

*Note:* Indicator loadings ≥ 0.70, composite reliability ≥ 0.70, and AVE ≥ 0.50 indicate satisfactory reflective measurement properties.

Subjective workload and job content were evaluated as formative constructs. Their indicators were assessed using outer weights, bootstrapping significance tests, and VIF values. For subjective workload, all formative indicators had statistically significant outer weights (*p* < 0.001). For job content, several indicators, including coworker support, isometric physical load, job skill, and supervisor support, had significant outer weights, whereas decision authority, job insecurity, physical activity and psychological job demands had nonsignificant outer weights. However, as shown in Table S2, these indicators had statistically significant outer loadings (*p* < 0.05). Consistent with PLS‐SEM guidelines, the nonsignificant indicators were retained because of their significant outer loadings and theoretical relevance to the multidimensional psychosocial work environment. The collinearity assessment indicated no critical multicollinearity among the formative indicators, with all outer VIF values well below the recommended threshold of 3.3 (Table 3).

**TABLE 3:** Formative measurement model assessment.

Construct	Indicator	Outer weight	STDEV	*t*‐value	*p*	VIF
Subjective workload	Mental demand	0.462	0.061	7.512	< 0.001	1.086
	Physical demand	0.343	0.055	6.189	< 0.001	1.028
	Temporal demand	0.440	0.056	7.855	< 0.001	1.026
	Performance	0.308	0.058	5.347	< 0.001	1.058
	Effort	0.286	0.059	4.831	< 0.001	1.092
	Frustration	0.375	0.073	5.127	< 0.001	1.028
Job content	Coworker support	0.286	0.070	4.068	< 0.001	1.217
	Decision authority	0.113	0.082	1.380	0.168	1.058
	Isometric physical load	0.379	0.085	4.463	< 0.001	1.326
	Job insecurity	−0.018	0.081	0.220	0.826	1.077
	Job skill	0.332	0.089	3.717	< 0.001	1.297
	Physical activity	0.119	0.070	1.707	0.088	1.182
	Psychological job demands	0.046	0.079	0.576	0.565	1.204
	Supervisor support	0.426	0.083	5.125	< 0.001	1.259

*Note:* Outer weights and their significance were obtained from bootstrapping. VIF values were obtained from the PLS‐SEM algorithm. *p*‐values reported as 0.000 in SmartPLS are presented as < 0.001. All VIF values were below 3.3, indicating no problematic collinearity among the formative indicators. Nonsignificant outer weights should be interpreted together with the corresponding outer loadings and theoretical relevance before considering indicator removal.

Discriminant validity among the reflective constructs was supported by the Fornell–Larcker criterion and HTMT assessment. The HTMT value between cognitive failure and job burnout was below the recommended threshold, indicating adequate discriminant validity (Table 4).

**TABLE 4:** Discriminant validity of reflective constructs.

Panel A. Fornell‐Larcker criterion		
Construct	Cognitive failure	Job burnout
Cognitive failure	0.982	
Job burnout	0.376	0.794

Panel B. Heterotrait‐monotrait ratio (HTMT)		
Construct	Cognitive failure	Job burnout
Cognitive failure	—	
Job burnout	0.419	—

*Note:* In Panel A, diagonal values represent the square root of the average variance extracted (AVE), and off‐diagonal values represent inter‐construct correlations. In Panel B, HTMT values below 0.85 indicate satisfactory discriminant validity.

### 3.2 Structural Model Assessment

**FIGURE 3:**
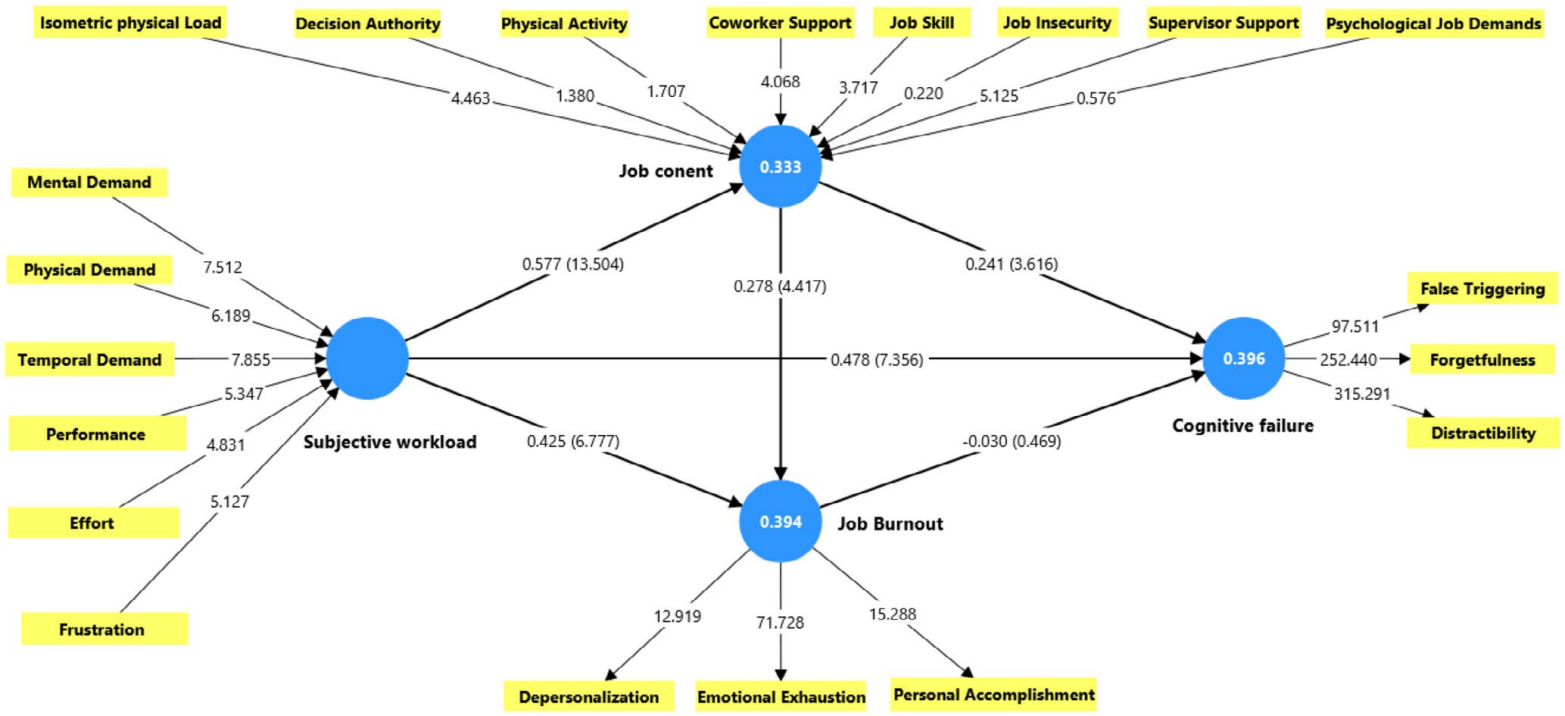
Direct model (*N* = 255).

The structural model results are presented in Figure 3 and Table 5. Subjective workload was significantly and positively associated with cognitive failure (*β* = 0.478, t = 7.356, *p* < 0.001), job burnout (*β* = 0.425, t = 6.777, *p* < 0.001), and job content (*β* = 0.577, t = 13.504, *p* < 0.001), indicating that higher workload is associated with a more unfavourable job content profile (i.e., higher demands and lower resources). A more unfavourable job content profile was also significantly associated with higher cognitive failure (*β* = 0.241, t = 3.616, *p* < 0.001) and higher job burnout (*β* = 0.278, t = 4.417, *p* < 0.001). In contrast, the association between job burnout and cognitive failure was not statistically significant (*β* = −0.030, t = 0.469, *p* = 0.639) (Table 5).

**TABLE 5:** Results of structural equation modelling.

Structural path	*β*	*t*‐value	*p*	95% CI Lower	95% CI Upper	Result
Subjective workload → Cognitive failure	0.478	7.356	< 0.001	0.350	0.606	Significant
Subjective workload → Job burnout	0.425	6.777	< 0.001	0.295	0.542	Significant
Subjective workload → Job content	0.577	13.504	< 0.001	0.505	0.670	Significant
Job content → Cognitive failure	0.241	3.616	< 0.001	0.110	0.373	Significant
Job content → Job burnout	0.278	4.417	< 0.001	0.157	0.405	Significant
Job burnout → Cognitive failure	−0.030	0.469	0.639	−0.164	0.086	Not significant

*Note:* *β* denotes the standardized path coefficient. Statistical significance was assessed using bootstrapping; *p* < 0.05 was considered statistically significant.

In addition to statistical significance, the practical relevance of the structural relationships was evaluated using explained variance (R^2^) and effect sizes (f^2^). The model explained 39.6% of the variance in cognitive failure, 39.4% of the variance in job burnout, and 33.3% of the variance in job content, indicating moderate explanatory power. Subjective workload demonstrated a large association with job content (f^2^ = 0.498) and moderate associations with cognitive failure (f^2^ = 0.211) and job burnout (f^2^ = 0.198). Job content showed small associations with cognitive failure (f^2^ = 0.059) and job burnout (f^2^ = 0.085), whereas job burnout showed a negligible association with cognitive failure (f^2^ = 0.001) (Table 6).

**TABLE 6:** Explanatory power and effect sizes of the structural model.

Endogenous construct/Structural path	*R* ^2^	f^2^	Interpretation
Cognitive failure	0.396	—	Moderate explanatory power
Job burnout	0.394	—	Moderate explanatory power
Job content	0.333	—	Moderate explanatory power
Subjective workload → Job content	—	0.498	Large effect
Subjective workload → Cognitive failure	—	0.211	Moderate effect
Subjective workload → Job burnout	—	0.198	Moderate effect
Job content → Cognitive failure	—	0.059	Small effect
Job content → Job burnout	—	0.085	Small effect
Job burnout → Cognitive failure	—	0.001	Negligible effect

*Note:* *R* ^2^ values indicate the proportion of variance explained in each endogenous construct. Effect sizes were interpreted as small (f^2^ = 0.02), medium (f^2^ = 0.15), and large (f^2^ = 0.35).

After adjustment for demographic and occupational characteristics, including age, work experience, shift work, and employment type, the main structural relationships remained stable. The adjusted model explained 42.3% of the variance in cognitive failure, 39.7% of the variance in job burnout, and 33.3% of the variance in job content. None of the included demographic or occupational covariates demonstrated statistically significant direct associations with cognitive failure or job burnout. The estimated effects of the control variables and the results of the adjusted structural model are presented in Table S3 and Figure S1.

### 3.3 Mediation Analysis

Bootstrapping analysis demonstrated a statistically significant indirect association between subjective workload and cognitive failure through job content (*β* = 0.139, t = 3.345, *p* = 0.001). Similarly, a statistically significant indirect association between subjective workload and job burnout through job content was observed (*β* = 0.160, t = 3.836, *p* < 0.001). In contrast, the indirect association between subjective workload and cognitive failure through job burnout was not statistically significant (*β* = −0.013, t = 0.464, *p* = 0.643) (Table 7).

**TABLE 7:** Results of mediation analysis.

Indirect pathway	Standardized Indirect effect (*β*)	*t*‐value	*p*	95% CI Lower	95% CI Upper	Result
Subjective workload → Job content → Cognitive failure	0.139	3.345	0.001	0.065	0.230	Significant
Subjective workload → Job burnout → Cognitive failure	−0.013	0.464	0.643	−0.072	0.037	Not significant
Subjective workload → Job content → Job burnout	0.160	3.836	< 0.001	0.089	0.253	Significant

*Note:* *β* denotes the standardized specific indirect effect. Statistical significance was assessed using bootstrapping; *p* < 0.05 was considered statistically significant.

### 3.4 Predictive Relevance and Model Fit

Predictive relevance was assessed using the Q^2^predict procedure. Positive Q^2^predict values were obtained for cognitive failure (Q^2^predict = 0.338), job burnout (Q^2^predict = 0.316), and job content (Q^2^predict = 0.285), indicating predictive relevance for all endogenous constructs.

Approximate model fit was evaluated using the standardized root mean square residual (SRMR), d_ULS, d_G, and the normed fit index (NFI). These indices were selected because they provide complementary information regarding the discrepancy between the observed and model‐implied correlation matrices and the comparative fit of the PLS‐SEM model. The NFI value was 0.750, indicating limited comparative fit. Bootstrap‐based exact‐fit assessment showed that SRMR (0.055) and d_ULS (0.640) exceeded their respective 95% and 99% upper bounds, whereas d_G (0.200) exceeded the 95% upper bound (0.197) but remained below the 99% upper bound (0.213). Thus, exact model fit was not fully supported, and model adequacy was interpreted cautiously by considering the R^2^ values, f^2^ effect sizes, Q^2^predict values and the magnitude and statistical significance of the structural paths.

## 4 Discussion

This study examined the association between subjective workload and cognitive failure and explored the indirect associations of job content and burnout among emergency nurses. The findings demonstrated that subjective workload was positively and significantly associated with cognitive failure, indicating that greater mental, physical and temporal demands were related to poorer cognitive functioning in the study sample. These results are consistent with previous studies reporting that increased workload is associated with deficits in memory, attention, and concentration (Jarahian Mohammady et al. 2018; Bufano et al. 2024). The demanding nature of emergency nursing, characterized by frequent interruptions, high patient acuity and rapid decision‐making, may contribute to the observed association between greater workload and increased cognitive failure (Surendran et al. 2024).

The observed statistically significant indirect association between subjective workload and cognitive failure through job content suggests that psychosocial work factors may be important in this relationship. In the present model, the job content construct was operationalized such that higher scores indicated a less favourable psychosocial work environment, characterized by higher job demands and lower job resources. Job content encompasses task complexity, autonomy, role clarity, physical demands and interpersonal support. Its formative specification reflects the multidimensional nature of the construct, in which distinct job demands and resources collectively characterize nurses' psychosocial work environment. Consistent with previous evidence (Mehri et al. 2022; Shan et al. 2023), a more unfavourable job content profile may be associated with greater cognitive strain, increased task demands, and reduced capacity to sustain attention and working memory. In emergency settings, where nurses perform complex tasks under severe time pressure, unfavourable job characteristics may be associated with greater cognitive overload and a higher reported likelihood of errors. These findings suggest that the observed association between workload and cognitive failure may reflect not only workload intensity but also nurses' perceptions of the broader psychosocial and structural characteristics of their work environment.

The present study found no evidence of a statistically significant indirect association between subjective workload and cognitive failure through job burnout. This non‐significant pathway represents one of the most notable findings of the study and warrants careful interpretation. One possible explanation is that, in emergency settings, the association between workload and cognitive functioning may reflect more immediate work experiences, whereas burnout represents a more chronic and cumulative response to occupational stress that may not parallel moment‐to‐moment cognitive failure in a cross‐sectional design. A second explanation is methodological. Because all variables were measured using self‐report questionnaires at a single time point, the observed relationships may have been influenced by differences in how participants perceived and reported acute workload experiences compared with longer‐term emotional exhaustion. A third explanation relates to measurement. Although burnout and cognitive failure are related constructs, they represent distinct dimensions of occupational functioning, and the instruments used to assess them capture different aspects of psychological strain. Burnout measures primarily assess chronic emotional and attitudinal responses to prolonged stress, whereas cognitive failure questionnaires evaluate perceived lapses in attention and memory. Consequently, the absence of a direct association may partly reflect differences in construct domains and measurement sensitivity. A fourth explanation is contextual. Emergency nurses work in fast‐paced, interruption‐rich environments in which acute operational demands may overshadow the measurable cognitive correlates of burnout. In contrast, burnout may demonstrate a stronger association with cognitive outcomes in inpatient or intensive care settings.

These findings are broadly consistent with recent evidence suggesting that cognitive burden among emergency nurses may be more closely related to real‐time workload conditions, whereas the association between burnout and cognitive burden may vary across clinical settings and measurement approaches. These findings are also consistent with broader psychological perspectives suggesting that responses to occupational stress may be associated with cognitive appraisal, coping strategies and psychological flexibility, rather than with burnout severity alone. Therefore, the absence of a statistically significant indirect association through job burnout should not be interpreted as evidence that burnout is unimportant. Instead, these findings suggest that the association between burnout and cognitive failure may be conditional, indirect, or more complex than originally hypothesized. Nevertheless, previous studies have reported significant associations between burnout and cognitive difficulties among healthcare professionals, suggesting that differences in organizational context, study design and participant characteristics may account for the variability in the reported associations between burnout and cognitive functioning (Cortés‐Álvarez et al. 2024; He et al. 2017). Although the model demonstrated predictive relevance for all endogenous constructs, the relatively low NFI (0.750) and bootstrap based exact fit results indicated limited model fit, reducing confidence in the proposed structural representation. Therefore, the observed direct and indirect associations should be interpreted cautiously and confirmed in future studies using independent samples and alternative model specifications.

In contrast, a statistically significant indirect association between subjective workload and job burnout through job content was observed. This finding is consistent with previous studies indicating that unfavourable job characteristics, including high psychological demands, limited autonomy and inadequate organizational support, are associated with emotional exhaustion and depersonalization (Gündüz and Öztürk 2025; Kuo et al. 2025). Accordingly, improvements in job design, participatory decision‐making, and supportive management practices may be relevant to efforts to reduce burnout risk and promote job satisfaction. Although some studies have reported no significant association between subjective workload and burnout (Barzani and Dal Yılmaz 2022), these inconsistencies may be related to differences in organizational culture, staffing patterns, workload distribution, measurement approaches and sample characteristics.

These findings should also be interpreted in light of their practical implications. Although subjective workload was significantly associated with nurses' cognitive functioning and job‐related outcomes, cognitive failure and burnout are multifactorial phenomena that are shaped by a wide range of individual and organizational factors. Recent literature indicates that broader work conditions, including extended working hours, irregular shift patterns, and high acuity departments, often interact with family demands and impair nurses' work‐life balance and overall well‐being (Sh 2026; Ghanim and Ibrahim 2026). Nurses facing high work demands and substantial family responsibilities have been reported to experience poorer work‐life balance, potentially increasing occupational strain and cognitive burden (Sh 2026; Ghanim and Ibrahim 2026). Organizational and social support, including flexible scheduling and spousal support, may help mitigate these challenges (Ghanim and Ibrahim 2026). Consequently, the findings suggest that addressing workload alone may not fully capture the factors associated with nurses' cognitive and psychological outcomes. Comprehensive workplace strategies targeting job design, organizational support and working conditions may be relevant considerations for improving these outcomes. Overall, these findings underscore the importance of addressing both job demands and job content as potential factors associated with cognitive functioning, burnout risk, and patient safety outcomes.

## 5 Limitations & Strengths

This study has several limitations. First, it was conducted exclusively among emergency nurses working in teaching hospitals, which may limit the generalizability of the findings to other clinical settings. Second, because the study used a cross‐sectional design, temporal ordering and causal relationships cannot be established, although the structural model tested directional hypotheses. Accordingly, all direct and indirect pathways should be interpreted as statistical associations rather than confirmed causal mechanisms. Third, the study relied exclusively on self‐report questionnaires, increasing the potential for common method variance, response bias and shared‐report inflation across constructs. Fourth, quota sampling is a non‐probability sampling method that may limit representativeness and increase the risk of selection bias. Moreover, although participants were recruited proportionately from five hospitals, the quota sampling structure was not explicitly incorporated into the statistical analyses. Consequently, hospital‐level clustering and differences in sampling fractions across centres may have influenced the estimated standard errors and further limited the generalizability of the findings. Fifth, the length and number of questionnaires may have increased participant burden and affected response accuracy. In addition, the demanding and unpredictable nature of emergency department work may have reduced participants' concentration while completing the questionnaires. Nonresponse bias is also possible, as nurses experiencing higher workload or burnout may have been less likely to complete the questionnaires, potentially leading to underestimation of these variables and their associations. To minimise these concerns, participants were provided with additional time and were encouraged to complete the questionnaires during periods of lower workload.

Another limitation is that the dataset was originally collected for a comprehensive assessment of emergency nurses and was previously used in an earlier publication to examine direct predictive associations (Parizad et al. 2026). However, the present study addresses a distinct research objective by testing a mediation framework using SEM. This secondary analysis enhances the use of the existing data without imposing additional burden on participants and provides distinct theoretical insights. In addition, several potentially relevant variables were not included in the model, including organizational climate, staffing adequacy, resilience, coping strategies, psychological flexibility, shift burden and other occupational and personal factors that may be associated with cognitive failure and burnout. Given the multifactorial nature of cognitive functioning among emergency healthcare professionals, future research should incorporate these variables, together with broader organizational and individual factors, to provide a more comprehensive understanding of the mechanisms underlying cognitive failure and burnout. Longitudinal or multi‐wave study designs are also needed to clarify temporal relationships and strengthen causal inference.

Despite these limitations, the study has several notable strengths. It simultaneously examined multiple interrelated variables, including subjective workload, job content, burnout and cognitive failure, providing a more comprehensive understanding of cognitive and psychological outcomes among emergency nurses. In addition, the use of SEM enabled the evaluation of complex direct and indirect relationships, thereby strengthening the methodological rigour of the study.

## 6 Conclusion

The findings indicate that job content demonstrated statistically significant indirect associations in the relationships between subjective workload and both cognitive failure and burnout among emergency nurses. However, burnout did not demonstrate a statistically significant indirect association between subjective workload and cognitive failure in the present sample. Emergency care is characterized by substantial mental, physical and psychological demands that may be associated with cognitive lapses and emotional strain among nurses.

Given these observed associations, healthcare managers and policymakers might consider strategies to optimize job design and address workload intensity. While these specific interventions were not directly evaluated in the present study, potential strategies such as optimizing nurse‐to‐patient ratios, increasing staffing levels, adopting digital technologies to streamline documentation, revising shift patterns warrant further investigation through targeted intervention studies. Future research should evaluate these approaches to determine their effectiveness in supporting nurses' cognitive functioning, reducing cognitive strain and burnout risk, and promoting safer patient care.

## 7 Implications for Nursing

The findings provide important insights for nursing managers seeking to support cognitive performance, reduce burnout, and contribute to improving patient safety in emergency care settings. Subjective workload and job content emerged as key factors associated with both cognitive failure and burnout, highlighting the potential value of managerial strategies that address organizational, structural, and psychosocial aspects of nursing work.

While this study did not directly evaluate specific workplace interventions, the observed associations suggest that workload optimization strategies, such as maintaining appropriate nurse‐to‐patient ratios, implementing strategic staffing practices and ensuring equitable distribution of complex tasks across shifts, warrant further investigation as potential avenues for support. Similarly, strategies aimed at reducing excessive time pressure, minimizing unnecessary interruptions and providing adequate rest periods could potentially support nurses' cognitive capacity during high‐intensity clinical activities, though these require empirical validation in interventional studies. Equally important is the potential optimization of job content and job design. Future research should evaluate whether improving role clarity, increasing decision‐making autonomy, strengthening interdisciplinary collaboration and providing supportive supervision effectively address the challenges associated with high workload. In addition, the potential role of skill development, competency‐based training and simulation‐based education in supporting nurses' preparedness for managing cognitively demanding clinical situations merits further exploration.

Additionally, nurse leaders might consider exploring systems for the early identification of burnout and cognitive strain through regular assessments, structured debriefings and reflective practice sessions. The provision of access to psychological support services, stress‐management programs and resilience‐building initiatives could potentially support a healthier and more sustainable work environment. Furthermore, the potential impact of investing in digital technologies, including smart documentation systems, clinical decision‐support tools and automated workload monitoring, to streamline clinical workflows and address cognitive burden associated with complex clinical activities should be rigorously evaluated in future intervention studies.

Overall, nursing managers play a critical role in fostering work environments that support nurse well‐being and address factors associated with cognitive failure and the quality and safety of patient care. By considering both workload intensity and job content, healthcare organizations may be better positioned to design, implement, and evaluate future strategies that support nursing performance, address staff retention challenges, and promote safer clinical environments for patients and healthcare professionals.

## Author Contributions

**Zahrasadat Abedi:** conceptualization, investigation, methodology, data curation, resources, project administration, formal analysis, validation, writing – review and editing, writing – original draft. **Keyvan Hosseingholipour:** data curation. **Parisa Nazmi:** writing – review and editing, data curation. **Naser Parizad:** conceptualization, investigation, funding acquisition, writing – original draft, writing – review and editing, methodology, formal analysis, data curation, supervision, validation, visualization, project administration, software, resources.

## Funding

The authors have nothing to report.

## Disclosure

This manuscript is based on a nursing research project titled “Investigating the Predictive Power of Job Content, Mental Workload, and Job Burnout on Cognitive Failures in Nurses Working in Emergency Departments of Urmia Hospitals in 2024” (Record No. 13143). The research was carried out by the first author, Zahrasadat Abedi, under the supervision of Professor Naser Parizad, with valuable guidance from advisors Keyvan Hosseingholipour and Parisa Nazmi, who assisted in designing and implementing the study. All authors have read and approved the final manuscript before submission.

## Conflicts of Interest

The authors declare no conflicts of interest.

## Supporting information


**Figure S1:** Structural model adjusted for demographic and occupational characteristics.

**Table S1:** STROBE Statement—Checklist of items that should be included in reports of *cross‐sectional studies*.

**Table S2:** Outer Loadings of Formative Constructs.

**Table S3:** Direct effects of demographic and occupational control variables.

## Data Availability

The data that support the findings of this study are available on request from the corresponding author. The data are not publicly available due to privacy or ethical restrictions.

## References

[nop270846-bib-0001] Abareshi, F. , F. Salami , F. Farina , et al. 2022. “The Impact of Mental Workload, Work‐Related and Socio‐Demographic Factors on Job Burnout Among Emergency Medical Staff.” Work 72: 1269–1277. 10.3233/WOR-210001. 35723141

[nop270846-bib-0002] Arthur, P. , B. Morrison , and J. K. Earl . 2025. “The Job Demand‐Resources (JD‐R) Model Through the Eyes of Financial Advisers: A Scoping Review.” Australian Journal of Management 51, no. 13: 959–983. 10.1177/03128962251350337.

[nop270846-bib-0003] Aschwanden, D. , A. R. Sutin , M. Luchetti , M. Allemand , Y. Stephan , and A. Terracciano . 2020. “A Systematic Review and Meta‐Analysis of the Association Between Personality and Cognitive Failures/Complaints.” Social and Personality Psychology Compass 14: e12565. 10.1111/spc3.12565. PMC8317966 34326894

[nop270846-bib-0004] Bakker, A. B. , and E. Demerouti . 2017. “Job Demands–Resources Theory: Taking Stock and Looking Forward.” Journal of Occupational Health Psychology 22: 273–285. 10.1037/ocp0000056. 27732008

[nop270846-bib-0005] Bakker, A. B. , E. Demerouti , and A. Sanz‐Vergel . 2023. “Job Demands–Resources Theory: Ten Years Later.” Annual Review of Organizational Psychology and Organizational Behavior 10: 25–53. 10.1146/annurev-orgpsych-120920-035819.

[nop270846-bib-0006] Bannerjee, A. , W. Elkins , S. M. Resnick , and M. Bilgel . 2025. “Alzheimer's Disease Neuropathology and Longitudinal Change in Subjective Cognitive Complaints.” Alzheimer's & Dementia: Diagnosis, Assessment & Disease Monitoring 17: e70176. 10.1002/dad2.70176. PMC12394183 40896725

[nop270846-bib-0007] Barzani, K. I. S. , and Ü. Dal Yılmaz . 2022. “The Relationship Between Mental Workload and Fatigue in Emergency Department Nurses.” Cyprus Journal of Medical Sciences 7: 381–386. 10.4274/cjms.2020.2039.

[nop270846-bib-0008] Broadbent, D. E. , P. F. Cooper , P. FitzGerald , and K. R. Parkes . 1982. “The Cognitive Failures Questionnaire (CFQ) and Its Correlates.” British Journal of Clinical Psychology 21: 1–16. 10.1111/j.2044-8260.1982.tb01421.x. 7126941

[nop270846-bib-0009] Bufano, P. , C. Di Tecco , A. Fattori , et al. 2024. “The Effects of Work on Cognitive Functions: A Systematic Review.” Frontiers in Psychology 15: 1351625. 10.3389/fpsyg.2024.1351625. PMC11112082 38784613

[nop270846-bib-0010] Choobineh, A. , H. Ghaem , and P. Ahmedinejad . 2011. “Validity and Reliability of the Persian (Farsi) Version of the Job Content Questionnaire: A Study Among Hospital Nurses.” Eastern Mediterranean Health Journal 17: 335–341. 22259893

[nop270846-bib-0011] Ciğerci, Y. , and E. P. Özpınar . 2026. “The Relationship Between Smartphone‐Related Distraction and Medical Error Tendency Among Surgical Nurses: A Cross‐Sectional Study.” BMC Nursing 25: 144. 10.1186/s12912-026-04292-w. PMC12888531 41535824

[nop270846-bib-0012] Cortés‐Álvarez, N. Y. , A. Lara‐Morales , E. Bautista‐Rodríguez , et al. 2024. “Job Burnout, Cognitive Functioning, and Brain‐Derived Neurotrophic Factor Expression Among Hospital Mexican Nurses.” PLoS One 19: e0304092. 10.1371/journal.pone.0304092. PMC11125546 38787900

[nop270846-bib-0013] Dall'Ora, C. , J. Ball , M. Reinius , et al. 2020. “Burnout in Nursing: A Theoretical Review.” Human Resources for Health 18: 41. 10.1186/s12960-020-00469-9. PMC7273381 32503559

[nop270846-bib-0014] Di Gesto, C. , G. R. Policardo , S. Bocci Benucci , E. Çela , and C. Grano . 2025. “Difficulties in Emotion Regulation and Stress in Intensive Care Unit Nurses During COVID‐19: Exploring the Mediating Role of Psychological Inflexibility and the Moderating Effect of Work Experience.” In Healthcare, vol. 13, No. 13, 1575. MDPI. 10.3390/healthcare13131575 PMC12249951 40648600

[nop270846-bib-0015] García‐Sierra, R. , J. Fernández‐Castro , and F. Martínez‐Zaragoza . 2016. “Relationship Between Job Demand and Burnout in Nurses: Does It Depend on Work Engagement?” Journal of Nursing Management 24: 780–788. 10.1111/jonm.12382. 27111251

[nop270846-bib-0016] Geraghty, S. , N. Narayan , and K. Oliver . 2025. “The Strain of Nursing Workloads on Patient Care.” Practice Nursing 36: 212–215. 10.12968/pnur.2025.0029.

[nop270846-bib-0017] Ghanim, R. S. , and R. H. Ibrahim . 2026. “Exploring Factors Affecting Work‐Life Balance Perceptions Among Female Nurses: A Cross‐Sectional Study in Mosul City Hospitals.” Malaysian Journal of Medicine & Health Sciences 22: 127. 10.47836/mjmhs.22.s3.19.

[nop270846-bib-0018] Gündüz, E. S. , and N. K. Öztürk . 2025. “Mental Workload as a Predictor of Burnout in Intensive Care Nurses.” Nursing in Critical Care 30: e13173. 10.1111/nicc.13173. PMC11877011 39420597

[nop270846-bib-0019] Hair, J. F., Jr. , G. T. M. Hult , C. M. Ringle , et al. 2021. Partial Least Squares Structural Equation Modeling (PLS‐SEM) Using R: A Workbook. Springer Nature.

[nop270846-bib-0020] Handel, M. J. 2017. “92Measuring Job Content: Skills, Technology, and Management Practices.” In The Oxford Handbook of Skills and Training, edited by J. Buchanan , D. Finegold , K. Mayhew , et al. Oxford University Press.

[nop270846-bib-0021] Hart, S. G. , and L. E. Staveland . 1988. “Development of NASA‐TLX (Task Load Index): Results of Empirical and Theoretical Research.” In Advances in Psychology, edited by P. A. Hancock and N. Meshkati , 139–183. North‐Holland.

[nop270846-bib-0022] He, S. , Y. Zhang , J. Zhan , et al. 2017. “Burnout and Cognitive Impairment: Associated With Serum BDNF in a Chinese Han Population.” Psychoneuroendocrinology 77: 236–243. 10.1016/j.psyneuen.2017.01.002. 28119229

[nop270846-bib-0023] Hitchcott, P. K. , M. C. Fastame , D. Langiu , and M. P. Penna . 2017. “Cognitive Failures in Late Adulthood: The Role of Age, Social Context and Depressive Symptoms.” PLoS One 12: e0189683. 10.1371/journal.pone.0189683 PMC5731702 29244847

[nop270846-bib-0024] Hoonakker, P. , P. Carayon , A. Gurses , et al. 2011. “Measuring Workload of ICU Nurses With A Questionnaire Survey: The Nasa Task Load Index (TLX).” IIE Trans Healthc Syst Eng 1: 131–143. 10.1080/19488300.2011.609524. PMC3388621 22773941

[nop270846-bib-0025] Jarahian Mohammady, M. , A. Sedighi , T. Khaleghdoost , et al. 2018. “Relationship Between Nurses' Subjective Workload and Occupational Cognitive Failure in Intensive Care Units.” Critical Care Nursing 11: 53–61.

[nop270846-bib-0026] Jin, T. , Y. Zhou , and L. Zhang . 2023. “Job Stressors and Burnout Among Clinical Nurses: A Moderated Mediation Model of Need for Recovery and Career Calling.” BMC Nursing 22: 388. 10.1186/s12912-023-01524-1. PMC10583433 37853383

[nop270846-bib-0027] Jun, J. , M. M. Ojemeni , R. Kalamani , J. Tong , and M. L. Crecelius . 2021. “Relationship Between Nurse Burnout, Patient and Organizational Outcomes: Systematic Review.” International Journal of Nursing Studies 119: 103933. 10.1016/j.ijnurstu.2021.103933. 33901940

[nop270846-bib-0028] Kakemam, E. , R. Kalhor , Z. Khakdel , et al. 2019. “Occupational Stress and Cognitive Failure of Nurses and Associations With on Self‐Reported Adverse Events: A National Cross‐Sectional Survey.” Journal of Advanced Nursing 75: 3609–3618. 10.1111/jan.14201Digital. 31531990

[nop270846-bib-0029] Karasek, R. , C. Brisson , N. Kawakami , I. Houtman , P. Bongers , and B. Amick . 1998. “The Job Content Questionnaire (JCQ): An Instrument for Internationally Comparative Assessments of Psychosocial Job Characteristics.” Journal of Occupational Health Psychology 3: 322–355. 10.1037//1076-8998.3.4.322. 9805280

[nop270846-bib-0030] Keykaleh, M. S. , H. Safarpour , S. Yousefian , et al. 2018. “The Relationship Between Nurse's Job Stress and Patient Safety.” Open Access Macedonian Journal of Medical Sciences 6: 2228–2232. 10.3889/oamjms.2018.389. PMC6290432 30559893

[nop270846-bib-0031] Khan, A. , D. Kim , R. Atwater , and R. Reddy . 2025. “Individual‐Focused Interventions for Physician Burnout: A Meta‐Analysis of Mindfulness, Coaching, and Peer Support.” Medicina 62: 39. 10.3390/medicina62010039. PMC12843167 41597325

[nop270846-bib-0032] Khanade, K. , and F. Sasangohar . 2017. “Stress, Fatigue, and Workload in Intensive Care Nursing: A Scoping Literature Review.” In Proceedings of the Human Factors and Ergonomics Society Annual Meeting, vol. 61, No. 1, 686–690. SAGE Publications.

[nop270846-bib-0033] Kuo, H.‐H. , C.‐C. Lan , H.‐W. Kuo , and P. Y. Lin . 2025. “Unraveling the Psychological Pathways Between Job Strain and Musculoskeletal Disorders: The Mediating Roles of Work‐Related Fatigue and Burnout Among Hospital Nurses.” Journal of Nursing Management 2025: 8819293. 10.1155/jonm/8819293. PMC12745052 41472868

[nop270846-bib-0034] Ma, J. , B. Lowndes , K. Chrouser , S. Hallbeck , and B. McCrory . 2020. “Developing a Subjective Instrument for Laparoscopic Surgical Workload in a High Fidelity Simulator Using the NASA‐TLX and SURG‐TLX.” IISE Transactions on Healthcare Systems Engineering 11: 161–169. 10.1080/24725579.2020.1854395.

[nop270846-bib-0035] Maslach, C. , S. Jackson , and M. Leiter . 1986. The Maslach Burnout Inventory. Consulting Psychologist Press.

[nop270846-bib-0036] Maslach, C. , W. B. Schaufeli , and M. P. Leiter . 2001. “Job Burnout.” Annual Review of Psychology 52: 397–422. 10.1146/annurev.psych.52.1.397. 11148311

[nop270846-bib-0037] Mehri, F. , A. Babaei‐pouya , and M. Karimollahi . 2022. “Intensive Care Unit Nurses in Iran: Occupational Cognitive Failures and Job Content.” Frontiers in Public Health 10: 786470. 10.3389/fpubh.2022.786470. PMC9099024 35570927

[nop270846-bib-0038] Moalemi, S. , Z. Kavosi , N. Beygi , et al. 2018. “Evaluation of the Persian Version of Maslach Burnout Inventory‐Human Services Survey Among Iranian Nurses: Validity and Reliability.” Galen Medical Journal 7: e995. 10.22086/gmj.v0i0.995. PMC8343696 34466422

[nop270846-bib-0039] Mohammadi, M. , J. Nasl Seraji , and H. Zeraati . 2013. “Developing and Accessing the Validity and Reliability of a Questionnaire to Assess the Mental Workload Among ICUs Nurses in One of the Tehran University of Medical Sciences Hospitals.” J Sch Public Health Inst Public Health Res 11: 87–96.

[nop270846-bib-0040] Paas, F. , and J. J. Van Merrienboer . 2020. “Cognitive‐Load Theory: Methods to Manage Working Memory Load in the Learning of Complex Tasks.” Current Directions in Psychological Science 29: 394–398. 10.1177/0963721420922183.

[nop270846-bib-0041] Parizad, N. , P. Nazmi , K. Hosseingholipour , and Z. Abedi . 2026. “The Predictive Power of Job Content, Subjective Workload, and Job Burnout on Cognitive Failures in Nurses Working in Emergency Departments.” International Emergency Nursing 85: 101746. 10.1016/j.ienj.2026.101746. 41689995

[nop270846-bib-0042] Pérez‐Fuentes, M. C. , M. M. Molero Jurado , A. B. Barragán Martín , et al. 2018. “The Mediating Role of Perceived Stress in the Relationship of Self‐Efficacy and Work Engagement in Nurses.” Journal of Clinical Medicine 8, no. 1: 10. 10.3390/jcm8010010. PMC6352085 30577596

[nop270846-bib-0043] Ren, Y. , G. Li , D. Pu , et al. 2024. “The Relationship Between Perceived Organizational Support and Burnout in Newly Graduated Nurses From Southwest China: The Chain Mediating Roles of Psychological Capital and Work Engagement.” BMC Nursing 23: 719. 10.1186/s12912-024-02386-x. PMC11459715 39379880

[nop270846-bib-0044] Rodziewicz, T. L. , and J. E. Hipskind . 2020. “Medical Error Prevention.” StatPearls Treasure Island (FL): StatPearls Publishing.

[nop270846-bib-0045] Sewell, J. L. , L. A. Maggio , O. Ten Cate , et al. 2019. “Cognitive Load Theory for Training Health Professionals in the Workplace: A BEME Review of Studies Among Diverse Professions: BEME Guide No. 53.” Medical Teacher 41: 256–270. 10.1080/0142159X.2018.1505034. 30328761

[nop270846-bib-0046] Sh, A. T. R. 2026. “Work Conditions, Family Demands, and Work–Life Balance Among Female Nurses in Mosul Hospitals.” Egyptian Journal of Occupational Medicine 50: 51–65. 10.21608/ejom.2026.460126.1395.

[nop270846-bib-0047] Shahgholian, M. 2024. “Psychometric Properties of Persian Version of Cognitive Failures Questionnaire.” Journal of Developmental Psychology Iranian Psychologists 19: 323–334.

[nop270846-bib-0048] Shan, Y. , J. Shang , Y. Yan , and X. Ye . 2023. “Workflow Interruption and Nurses' Mental Workload in Electronic Health Record Tasks: An Observational Study.” BMC Nursing 22: 63. 10.1186/s12912-023-01209-9. PMC9996908 36890555

[nop270846-bib-0049] Søvold, L. E. , J. A. Naslund , A. A. Kousoulis , et al. 2021. “Prioritizing the Mental Health and Well‐Being of Healthcare Workers: An Urgent Global Public Health Priority.” Frontiers in Public Health 9: 679397. 10.3389/fpubh.2021.679397. PMC8137852 34026720

[nop270846-bib-0050] Surendran, A. , L. Beccaria , and P. McIlveen . 2025. “Cognitive Burden in Emergency Care: A Qualitative Exploratory Study of Emergency Nurses.” Journal of Nursing Management 2025: 6332434. 10.1155/jonm/6332434. PMC12682449 41362590

[nop270846-bib-0051] Surendran, A. , L. Beccaria , S. Rees , and P. Mcilveen . 2024. “Cognitive Mental Workload of Emergency Nursing: A Scoping Review.” Nursing Open 11: e2111. 10.1002/nop2.2111. PMC10873679 38366782

[nop270846-bib-0052] Sweller, J. , J. J. Van Merriënboer , and F. Paas . 2019. “Cognitive Architecture and Instructional Design: 20 Years Later.” Educational Psychology Review 31: 261–292. 10.1007/s10648-019-09465-5.

[nop270846-bib-0053] Tubbs‐Cooley, H. L. , C. A. Mara , A. C. Carle , B. A. Mark , and R. H. Pickler . 2019. “Association of Nurse Workload With Missed Nursing Care in the Neonatal Intensive Care Unit.” JAMA Pediatrics 173: 44–51. 10.1001/jamapediatrics.2018.3619. PMC6583427 30419138

[nop270846-bib-0054] Wallace, J. C. 2004. “Confirmatory Factor Analysis of the Cognitive Failures Questionnaire: Evidence for Dimensionality and Construct Validity.” Personality and Individual Differences 37: 307–324. 10.1016/j.paid.2003.09.005.

[nop270846-bib-0055] Wong, K. P. , B. Zhang , Y. J. Xie , et al. 2024. “Impacts of Job Demands on Turnover Intention Among Registered Nurses in Hong Kong Public Hospitals: Exploring the Mediating Role of Burnout and Moderating Effect of Pay Level Satisfaction.” Journal of Nursing Management 2024: 3534750. 10.1155/2024/3534750. PMC11918518 40224872

[nop270846-bib-0056] Xiong, Q. , F. Luo , Y. Chen , et al. 2024. “Factors Influencing Fatigue, Mental Workload and Burnout Among Chinese Health Care Workers During Public Emergencies: An Online Cross‐Sectional Study.” BMC Nursing 23: 428. 10.1186/s12912-024-02070-0. PMC11197284 38918772

[nop270846-bib-0057] Yuan, Z. , J. Wang , F. Feng , et al. 2023. “The Levels and Related Factors of Mental Workload Among Nurses: A Systematic Review and Meta‐Analysis.” International Journal of Nursing Practice 5: e13148. 10.1111/ijn.13148. 36950781

[nop270846-bib-0058] Zanabazar, A. , and S. Jigjiddorj . 2022. “Relationships Between Mental Workload, Job Burnout, and Organizational Commitment. In: *SHS Web of Conferences* 2022, 01003.” EDP Sciences 132: 01003.

