# Supplementary material for: Subjective Workload and Cognitive Failure Among Emergency Nurses: A Cross‐Sectional Mediation Analysis of Job Content and Job Burnout
Source: Nurs Open. 2026 Sep 21;13(9):e70846. doi: 10.1002/nop2.70846 (PMC13594400; doi:10.1002/nop2.70846)
Supplement: Supplementary file 1 [file NOP2-13-e70846-s002.docx]

# Supplementary Table S3. Direct effects of demographic and occupational control variables

| **Path** | **Original sample (β)** | **t-value** | **p-value** | **Interpretation** |
| --- | --- | --- | --- | --- |
| Age → Cognitive failure | -0.181 | 1.165 | 0.244 | Not significant |
| Age → Job Burnout | -0.085 | 0.536 | 0.592 | Not significant |
| Experience → Cognitive failure | 0.077 | 0.496 | 0.620 | Not significant |
| Experience → Job Burnout | 0.089 | 0.546 | 0.585 | Not significant |
| Shift work → Cognitive failure | 0.053 | 0.388 | 0.698 | Not significant |
| Shift work → Job Burnout | -0.025 | 0.162 | 0.872 | Not significant |
| Contractual employment_ → Cognitive failure | -0.186 | 1.312 | 0.190 | Not significant |
| Contractual employment → Job Burnout | -0.161 | 1.249 | 0.212 | Not significant |
| Temporary employment → Cognitive failure | 0.068 | 0.463 | 0.644 | Not significant |
| Temporary employment → Job Burnout | -0.082 | 0.622 | 0.534 | Not significant |

Note. Control variables were incorporated into the adjusted PLS-SEM model to examine their direct associations with cognitive failure and job burnout. β represents the standardized path coefficient.
