# Supplementary material for: Subjective Workload and Cognitive Failure Among Emergency Nurses: A Cross‐Sectional Mediation Analysis of Job Content and Job Burnout
Source: Nurs Open. 2026 Sep 21;13(9):e70846. doi: 10.1002/nop2.70846 (PMC13594400; doi:10.1002/nop2.70846)
Supplement: Supplementary file 2 [file NOP2-13-e70846-s003.docx]

Supplementary Figure S1. Structural model adjusted for demographic and occupational characteristics
