# Supplementary material for: Subjective Workload and Cognitive Failure Among Emergency Nurses: A Cross‐Sectional Mediation Analysis of Job Content and Job Burnout
Source: Nurs Open. 2026 Sep 21;13(9):e70846. doi: 10.1002/nop2.70846 (PMC13594400; doi:10.1002/nop2.70846)
Supplement: Supplementary file 3 [file NOP2-13-e70846-s004.docx]

# **Supplementary Table S2. Outer Loadings of Formative Constructs**

| **Construct** | **Indicator** | **Outer Loading** | **Sample Mean** | **STDEV** | **T Statistics** | **P Value** |
| --- | --- | --- | --- | --- | --- | --- |
| **Job Content** | Coworker support | 0.563 | 0.561 | 0.074 | 7.579 | <0.001 |
|  | Decision authority | 0.236 | 0.233 | 0.105 | 2.262 | 0.024 |
|  | Isometric physical load | 0.691 | 0.690 | 0.046 | 15.139 | <0.001 |
|  | Job insecurity | 0.329 | 0.325 | 0.082 | 4.001 | <0.001 |
|  | Job skill | 0.627 | 0.619 | 0.053 | 11.894 | <0.001 |
|  | Physical activity | 0.438 | 0.470 | 0.166 | 2.640 | 0.008 |
|  | Psychological job demands | 0.437 | 0.432 | 0.070 | 6.269 | <0.001 |
|  | Supervisor support | 0.602 | 0.603 | 0.054 | 11.111 | <0.001 |
| **Subjective Workload** | Mental demand | 0.544 | 0.544 | 0.059 | 9.178 | <0.001 |
|  | Physical demand | 0.357 | 0.353 | 0.073 | 4.869 | <0.001 |
|  | Temporal demand | 0.450 | 0.446 | 0.072 | 6.267 | <0.001 |
|  | Performance | 0.374 | 0.370 | 0.064 | 5.880 | <0.001 |
|  | Effort | 0.507 | 0.501 | 0.060 | 8.427 | <0.001 |
|  | Frustration | 0.448 | 0.443 | 0.061 | 7.326 | <0.001 |
| **Job Burnout** | Emotional exhaustion | 0.884 | 0.886 | 0.012 | 71.003 | <0.001 |
|  | Depersonalization | 0.736 | 0.732 | 0.048 | 15.452 | <0.001 |
|  | Reduced personal accomplishment | 0.755 | 0.753 | 0.057 | 13.205 | <0.001 |
| **Cognitive Failure** | Distractibility | 0.982 | 0.982 | 0.003 | 313.814 | <0.001 |
|  | False triggering | 0.978 | 0.979 | 0.010 | 97.855 | <0.001 |
|  | Forgetfulness | 0.985 | 0.985 | 0.004 | 251.176 | <0.001 |

Note: Outer loadings and their significance were obtained from bootstrapping procedure (5,000 subsamples). All outer loadings are statistically significant (p < 0.05), supporting the retention of all formative indicators despite some non-significant outer weights.
